# Preventive family education in a transitional welfare context: public perceptions, institutional capacity, and gender perspectives in North Macedonia

**DOI:** 10.3389/fsoc.2026.1860995

**Published:** 2026-09-22

**Authors:** Keskinova Angelka, Makedonka Radulovic, Irena Avirovic-Bundalevska, Stefan Vasev, Elena Chekova, Ivana Gegoska, Dushanka Petrova, Alenka Binicki Arsenovski, Nevena Petrovska

**Affiliations:** 1 Faculty of Philosophy, University Ss. Cyril and Methodius, Skopje, Republic of North Macedonia; 2 Institute for Social Affairs, Skopje, Republic of North Macedonia

**Keywords:** family education, marital stability, prevention, republic of North Macedonia, social policy

## Abstract

Family education (FE) is a preventive measure aimed at maintaining and promoting family stability throughout all family life cycles. The aim of our research was to examine the survey participants’ perceptions of the importance and availability of FE, and to assess the actual capacity of relevant institutions to implement FE programs. We used a quantitative approach through an online questionnaire, combined with a mapping of institutions offering FE programs. The sample consisted of 514 respondents and 20 institutions that offered family support. The data were analyzed using descriptive statistics and Cronbach’s alpha to assess reliability and chi-square to examine differences in the gender perspective of FE. Survey respondents expressed broad support for FE as a preventive measure, especially before marriage, as well as maintaining healthy family communication and relational stability. However, respondents and the mapped institutions indicate very low institutional support and a lack of FE programs. The findings indicate that although FE is highly accepted among the surveyed respondents, its integration into social policy and the institutional system remains limited. Currently, its function is directed reactively towards minimizing the consequences of already existing dysfunctional patterns in the family, instead of developing and maintaining its preventive function.

## 1 Introduction

The beginnings of family education (FE) date back to the early 20th century, first through marriage education and family economics movements (Stage and Vincenti, 1997), and later through professional organizations that institutionalized family education by setting standards and norms (Arcus et al., 1993). In the 1970s, FE spread throughout Europe through state-level preventive social policies (Duncan and Goddard, 2017). Gradually, it evolved into an evidence-based preventive discipline that has a significant role in ensuring quality family life and promoting family well-being across the lifespan.

Family life education (FE) is a preventive educational process intended to foster individual and family development across the lifespan by increasing knowledge, skills, and relational competencies [National Council on Family Relations (NCFR), 2019; Darling et al., 2021]. FE can be defined in many ways, depending on the purpose, content, and specific audience. In general, all definitions have three foundations: addressing problems influencing families, preventing problems, and developing the potential of individuals and families. Terms such as prevention/education/collaboration best describe FE (Радуловиќ и др., 2024).

The NCFR identifies the following as the most important factors for maintaining healthy family relationships: strong communication skills, knowledge of typical human development, sound decision-making, positive self-esteem, and healthy interpersonal relationships. For a given content to be characterized as family education, it should meet the following criteria: (1) It addresses individuals and families across the life cycle; (2) It is based on the needs of individuals and families; (3) It has a multidisciplinary theoretical approach and a multiprofessional practical approach, (4) It has an educational rather than a therapeutic approach; (5) It advocates and recognizes diverse family values; (6) It involves qualified professionals who know families professionally [National Council on Family Relations (NCFR), 2019].

The term family education (FE) is used throughout this paper as an umbrella term that covers preventive educational programs: premarital education, parental education, and relationship education. Counseling services are discussed separately as institutional interventions that may complement, but are not synonymous with, FE.

Global research confirms the positive impact of FE on family functioning. Hawkins et al. (2008), in their meta-study, confirm the positive impact of marriage and relationship education on relationship quality and communication skills. A meta-analysis of 17 published papers found that at least 14 reported improved marital satisfaction following a relationship education program (Halford and Bodenmann, 2013).

A similar analysis was conducted through the Healthy Marriage Initiative in the United States in the early 20th century, which included marriage and relationship education for low-income couples. They observed a small to moderate but consistent improvement in communication and relationship skills. They also conclude that couples at moderate risk benefit more than those at severe risk, and that more engaged (with higher program attendance) couples develop more practical skills and greater marital satisfaction (Wood et al., 2014).

Unlike in the USA, FE in Europe is not institutionalized in the same way; there are no centralized family education centers, but these programs are vital to public health care systems, social services, and early intervention systems. Analysis of European family education programs shows that they are most often universal (aimed at all families), selective (aimed at families at risk), or indicated (aimed at early signs of family dysfunction) (Gordon, 1983; Arnold et al., 2020). The programs most often focus on communication skills, dyadic coping, co-parenting quality, conflict resolution, and attachment-based emotional intervention (Bodenmann et al., 2011; Johnson, 2004).

Despite differences in the systematization of FE programs across Europe, research results still highlight their positive effects. The longitudinal study by Hahlweg et al. (1998) examined the effects of premarital and early communication skills, resulting in considerable advances in constructive communication, reduced rates of couples’ dissolution, and greater relationship satisfaction compared with a control group. Emotion-focused therapy, which is widely used in countries across Europe, especially the UK, Sweden, Norway, and the Netherlands, has shown strong effects on attachment security in the relationship, with 70% of couples transitioning more easily from distress to recovery, and has provided positive longitudinal effects measured over 2 years (Wiebe and Johnson, 2016).

Previous research clearly points to a direct connection between gender and family functioning: building family relationships, caring for family members, and participating in FE programs. The results position women as dominant in providing emotional regulation, building communication ties within the family, and seeking professional support, whereas men show less motivation to seek and participate in preventive family services (Hochschild, 1983; Coltrane, 2000; Addis and Mahalik, 2003). These findings are particularly relevant in transitional welfare contexts, where traditionally divided roles strongly affect the organization and functioning of the family system. Hence, examining gender differences is especially relevant when analyzing FE.

The Macedonian family has gone through considerable changes over the last 30 years. These changes are due to a shift in the traditional understanding of the family, a decrease in the number of family members, a decrease in families with children, and, in turn, an increase in single-person and single-parent families (Radulović et al., 2024). Statistical indicators from the 2021 census highlight important demographic changes that further change family dynamics, such as a decrease and aging of the population, further a continuous increase in the number of divorces which, according to the latest data in 2023, is 9.4% higher than in 2022, delaying marriage and a continuous increase in the age of partners entering into their first marriage, to 27.5 years for girls and 30.1 years for men, data relevant for 2023 (State Statistical Office, 2023). Radulović et al. (2024) examine young people’s perceptions and how they change over 8 years, from 2018 to 2022. The main conclusions confirm the abandonment of traditional family forms and the acceptance of the nuclear family, but the fact that, over the years, young people have reported a deterioration in family relationships, increased conflicts, and communication problems within the family is concerning. FЕ is a relatively new concept in the Republic of North Macedonia (RNM), emerging in response to major changes within the family and, from a demographic perspective, to demographic trends. FE as a system remains in its infancy, seeking to institutionalize itself and become part of social systems. In this regard, FE represents a potential opportunity to transition from a crisis-oriented to a prevention-oriented model of support, with the focus shifting from reducing consequences to building perspectives and communication skills before serious disruptions in family life and dynamics occur. In conditions of accelerated demographic and sociocultural changes, FE needs to be integrated into social policies and institutional practices.

The analysis of the state of the services provided by employees in the Social Work Centers (SWCs) indicates serious challenges in the treatment of marriage and family. Table 1 provides an overview of the number of services arranged annually from 2020 to 2023. The analysis of the table clearly shows that every year we have an increase in the services provided by the SWCs; of particular concern is the increase in divorce counseling, arrangement of personal relationships, and direct contact between a child and a parent/close relative (which is carried out at the request of the court as a legislative body), as well as deprivation of parental rights. The authors of this analysis concluded that dysfunctional family patterns are on the rise, particularly domestic violence, while SWCs face shortages of professional staff, inadequate spatial conditions, and limited professional development to deal with complex family problems (Бинички Арсеновски, 2024; Бинички Арсеновски et al., 2024).

**Table 1:** Number of services provided by CSWs in the period from 2020 to 2023.

Services from CSWs	2020 year	2021 year	2022 year	2023 year
Preparation for marriage, family life, parenting, family planning, and marital counseling for partners with troubled relationships	736	784	839	902
Divorce counseling	1,570	1,806	1,727	2,514
Professional help and support for broken partner/marital/family relationships	347	567	612	768
Peaceful procedure and entrustment of a child	2,081	2,373	2,307	2,585
Arrangement of personal relationships and direct contact between a child and a parent/close relative	1,421	2,027	2,510	2,743
Deprivation of parental rights	18	34	34	54

Premarital counseling, as a form of FE, was mandatory in the RNM until 2007, when, due to workload, the SWCs were forced to discontinue this activity. According to the data, 21 marriage and family counseling centers should operate nationwide and, among other services, they should offer premarital counseling, either at the request of service users or in cases where a minor seeks to marry (Советувалиште за брак и семејство, 2025). Table 1 presents data on the increase in the number of this type of counseling, which, in 2023, reached a maximum of 902 counseling services (Бинички Арсеновски et al., 2024).

According to the Law on Social Protection (2019), Article 73 defines the counseling service and the aspects it covers: preparation for marriage, family life, parenthood, family planning, marriage/partner counseling for partners with disrupted relationships, divorce counseling, family mediation for families with disrupted family relationships, psychosocial support for victims of domestic violence, psychosocial treatment of perpetrators of domestic violence and other specialized types of counseling. The adoption of the law also begins the process of licensing social service providers, allowing social service providers to be institutions outside the state. With the amendment of the law in 2025, for the first time, family educator was included as a professional profile within the social welfare system. This marks the first steps towards providing institutional support to FE.

Although numerous international studies clearly demonstrate the effectiveness of FE programs in strengthening the family system, as well as their preventive function in later family difficulties, research rarely focuses on the institutional system’s readiness and devotion to respond to these needs. This gap between community perceptions and institutional readiness is notably visible in transitional contexts where preventive family services are still developing, and institutional structures remain fragmented. Through integrating respondents’ perceptions with institutional mapping, the present study moves beyond evaluating attitudes towards family education and examines the extent to which social demand is aligned with existing institutional capacity. This approach gives a more comprehensive understanding of the opportunities and challenges involved in institutionalizing family education as a preventive social policy.

Accordingly, this study aims to examine respondents’ perceptions of FE in the RNM and to assess the institutional capacity for its implementation. By combining survey data from the survey respondents with institutional mapping, the study attempts to identify possible differences between demand for FE and the availability of institutional support.

## 2 Materials and methods

### 2.1 Research design and sampling

The study aimed to examine the survey participants’ perceptions of the importance and availability of FE, particularly as a preventive measure before marriage and as a means of supporting family functioning, and to assess the actual capacity of relevant institutions to implement FE programs. This study employed a cross-sectional quantitative design complemented by descriptive institutional mapping. The mapping component was not qualitative in nature but focused on structured descriptive data collection.

In this regard, the study consists of two components:

(1) *Analysis of the survey participants’ perceptions -* Data collection was carried out over 2 months, from October to November 2025. The sample is convenience-based and comprises 514 respondents. The demographic structure of the sample is as follows: women comprise 435 (84.63%) and men 79 (15.37%). According to the age distribution, 85 participants (16.53%) were aged 18–29 years, 262 (50.97%) were aged 30–44 years, 154 (29.96%) were aged 45–59 years, and 13 respondents (2.54%) were aged 60 or older. In terms of marital status, 364 of the respondents were married (70.82%), 57 were in a relationship (11.09%), 51 were single (9.92%), 27 were divorced (5.25%), 8 were widowed (1.56%), and 7 were in a cohabiting union (1.36%). By level of education, 307 participants had higher (undergraduate) education (59.73%), followed by 136 respondents with postgraduate education or higher (26.46%), 70 with secondary education (13.62%), and 1 with primary education or lower (0.19%). Figure 1 presents the demographic characteristics of the study participants.
(2) Mapping of institutions offering FE in the RNM was carried out using a specially created questionnaire. Institutions were considered to offer FE if they provided structured educational, counseling, preventive, parenting, premarital, marital, or family-support services aimed at strengthening family functioning in accordance with the Social Protection Law or their registered activities. The questionnaire was distributed to all 30 State Social Work Centers, which constitute the complete public network responsible for family-related social services in the RNM, as well as to 20 private associations that, according to the national registry, had statutory activities including work with children, parents, or families. The research lasted 4 months, from October 2025 to January 2026, after which we received feedback from 20 institutions (15 Social Work Centers, with a response rate of 50%, and 5 private associations, with a response rate of 25%).

**Figure 1:**
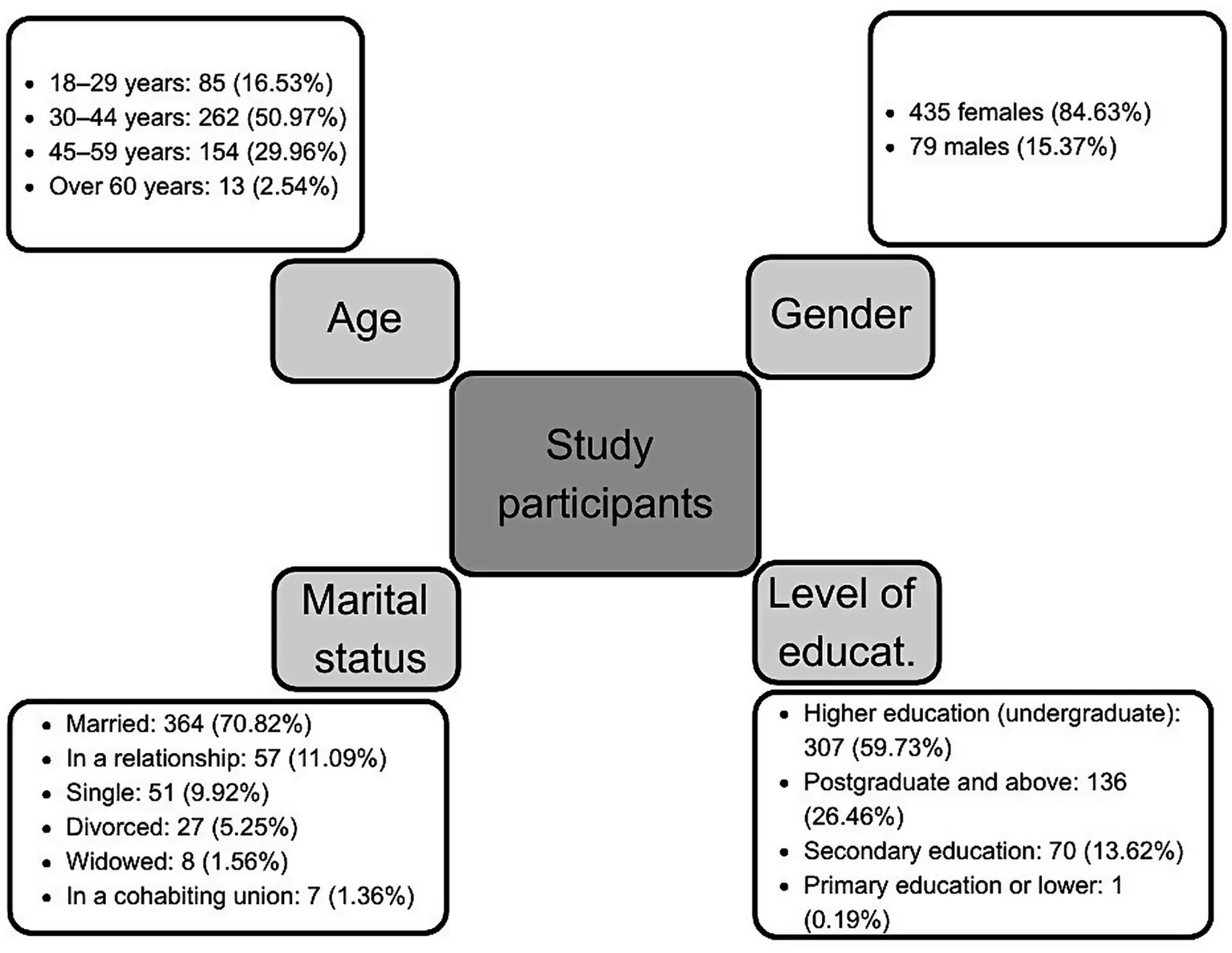
Demographic characteristics of participants (*n* = 514).

### 2.2 Instrument, procedure, and data analysis

We analyzed the survey participants’ perceptions using a custom-designed questionnaire following an extensive review of the FE literature (National Council on Family Relations (NCFR), 2019; Darling et al., 2021; Duncan and Goddard, 2017).

In general, we divided the questions into 4 segments that assessed:

- The importance of FE;
- The availability of FE;
- Priorities among different groups and
- Barriers to seeking FE before divorce.

The items were measured using a Likert-type scale (from 1 – strongly disagree to 5 strongly agree).

It was distributed online via the KoboToolBox platform.

While the mapping of institutions was carried out with a semi-structured questionnaire in Google Forms, it was composed of 20 open and closed questions, with which we assessed:

- Characteristics and activities of the institution,
- Involvement in the implementation of FE programs
- Their experience in terms of priorities and barriers in obtaining and providing programs for FE.

In both questionnaires, most items were specifically developed for this study based on the FE literature and national context because no existing instrument addressed all dimensions under investigation. To ensure content validity, the preliminary version of the questionnaires was reviewed by five experts: two in family studies, two social workers, and one pedagogist. Based on their recommendations, several items were revised to improve clarity, relevance, and conceptual coverage. The questionnaires were not formally pilot-tested before data collection.

The collected data were analyzed using IBM SPSS Statistics. Descriptive statistics were used to examine the respondents’ central tendencies and variability. We calculated the following statistical indicators: percentage (%), Arithmetic Mean (M), Standard Deviation (SD), and the Standard Error of the Mean (SEM) to indicate the precision of the estimated mean. Cronbach’s alpha was used to assess the internal consistency of the measurement scales. To analyze the gender perspective of FE, we used the chi-square test at a significance level of *p* < 0.05. Chi-square analysis was selected because the variables examined were categorical and the objective was to test associations between gender and responses.

Given the predominance of women and respondents with higher education in the sample, inferential findings should be interpreted as exploratory rather than representative of the wider population.

## 3 Results

### 3.1 The importance of FE

The results indicate that respondents perceive FE as important, *w*ith particular emphasis on its preventive role (Table 2). This perception is reflected in the high rating of the importance of premarital FE (*M* = 4.37, SD = 0.85, SEM = 0.03), respondents’ perceptions of the potential role of FE in preserving marital harmony and preventing problems (*M* = 4.16, SD = 0.84, SEM = 0.03), as well as its perceived role in supporting good marital communication (*M* = 4.29, SD = 0.79, SEM = 0.03). Respondents generally agree with the statement that attending FE may reduce the risk of divorce (*M* = 3.97, SD = 0.91, SEM = 0.04).

**Table 2:** Perception of the importance of FE.

Statement	*M*	SD	SEM	Cronbach’s *α*
FE is important for young couples before marriage	4.37	0.85	0.03	0.91
FE can be an effective preventive measure for avoiding problems in marriage	4.16	0.84	0.03	
FE can help preserve marriage by improving communication	4.29	0.79	0.03	
If couples attend FE, it will reduce the risk of divorce	3.97	0.91	0.04	
FE can make divorce easier by preparing couples for a peaceful separation	4.1	0.81	0.03	
FE should focus more on preventing divorce than on facilitating it	3.82	1.12	0.04	

Respondents also attributed greater importance to the preventive role of FE in supporting stable marital relationships (*M* = 3.82, SD = 1.12, SEM = 0.04) rather than facilitating divorce in the marriage system. However, the high SD indicates substantial variability in the answers. Respondents also expressed strong agreement with the view that, in the event of divorce, FE may facilitate a more peaceful separation of spouses (*M* = 4.1, SD = 0.81, SEM = 0.03). Hence, the results indicate that, in addition to emphasizing the preventive role of FE, which they regard as fundamental, respondents also perceive FE as potentially useful when marital problems arise.

The analysis of variability showed a high level of internal consistency (Cronbach’s *α* = 0.91) (Cronbach, 1951), indicating high internal consistency of the scale measuring the preventive and supportive role of FE.

According to the mapping, despite the recognition of the preventive role of FE, the experience of professionals shows that their help is sought when there is already a serious problem in the family system (Figure 2): that is, in 40% of cases, partners seek counseling during divorce, 30% counseling during a problem in the parental system, 8.5% of parents seek help in entrusting children in the process of divorce, and the same percentage counseling in cases of domestic violence. FE as a preventive measure accounts for 13%, of which 8.5% is marital, and 4.5% is premarital.

**Figure 2:**
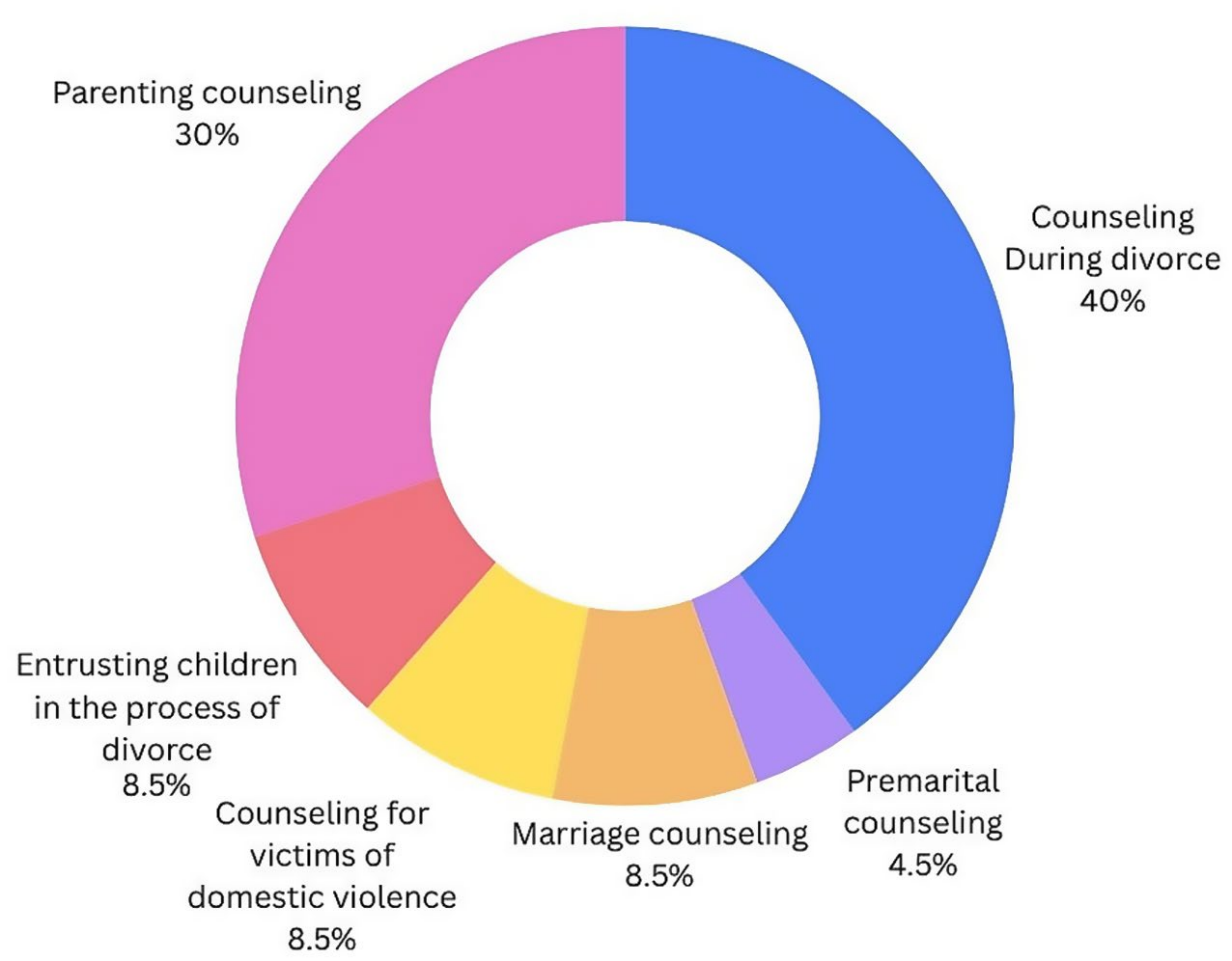
The most common reasons why couples seek professional support.

### 3.2 The availability of FE

The analysis of the results on the availability of FE programs in the RNM showed low agreement among respondents (Table 3). Most respondents reported an insufficient number of FE programs (*M* = 2.6, SD = 1.22, SEM = 0.05). Similar results were obtained regarding the perceived availability of programs, where a low mean score, combined with moderate variability, indicates that respondents perceive FE programs as insufficiently accessible, visible, or unevenly distributed (*M* = 2.57, SD = 1.28, SEM = 0.05).

**Table 3:** Perception of the availability of FE (survey participants’ perceptions).

Statement	*M*	SD	SEM	Cronbach’s *α*
FE programs are offered at RNM	2.6	1.22	0.05	0.70
FE is available to the general population in my community.	2.57	1.28	0.05	
The media and institutions in the RNM sufficiently promote FE as a preventive measure	2.04	1.09	0.04	

The lowest level of agreement was observed regarding the promotion of FE in the media and institutions as a preventive measure (*M* = 2.04, SD = 1.09, SEM = 0.04), indicating untapped potential to raise awareness, build institutional support, and promote FE systemically.

These findings indicate that respondents perceive substantial limitations in the availability and promotion of FE.

The reliability coefficient for this set of items (Cronbach’s *α* = 0.70) (Cronbach, 1951) indicates an acceptable level of internal consistency, which indicates reasonable and acceptable consistency for exploratory research.

The mapping of centers offering FE services (provide counseling services in accordance with Article 73 of the Social Protection Law) corroborates the respondents’ perception of the availability of FE programs. According to the mapping results, there is officially only one state family counseling center, represented by four counselors, and 4 NGOs that are licensed social service providers or are in the process of licensing, which offer family counseling services. All of them are located in the capital, thereby centralizing FE and limiting it to a particular group. The rest of the mapped institutions are represented by Centers for Social Work, one center in each city, which work with families in the process of divorce, entrustment of minors, and parenting after divorce. While CSWs provide legally mandated family-related social services, the state counseling center provides specialized counseling services under the Social Protection Law, whereas NGOs provide licensed and project-based preventive FE services (Table 4).

**Table 4:** Characteristics of institutions included in the institutional mapping of FE services in the RNM.

Institution type	No. of respondents	Main services	Geographic coverage
Centers for Social Work	15	Divorce, custody, parenting after divorce	Nationwide
State counseling center	1	Parenthood, marriage/partner counseling for partners with disrupted relationships, divorce counseling, psychosocial support for victims of domestic violence	Skopje (capital city)
NGOs	4		

### 3.3 Priorities among different groups

Respondents agree on the importance of FE, but there is no conviction regarding whether a certain group has an advantage or a greater need to use FE programs (Table 5). In this group of questions, we analyzed rural–urban differences (*M* = 2.47, SD = 1.35, SEM = 0.06), gender (*M* = 2.61, SD = 1.36, SEM = 0.06), and age (*M* = 2.67, SD = 1.19, SEM = 0.05).

**Table 5:** Perception of the priorities for FE among different groups (survey participants’ perceptions).

Statement	*M*	SD	SEM	Cronbach’s *α*
People with higher levels of education have less need for FE	2.1	1.23	0.05	0.87
In rural areas, FE has greater importance than in urban areas	2.47	1.35	0.06	
Men have a greater need for FE than women	2.61	1.36	0.06	
The importance of FE increases with age	2.67	1.19	0.05	

The high reliability value (Cronbach’s *α* = 0.87) indicates a high level of internal consistency, suggesting a coherent scale for measuring perceptions of prioritizing a particular demographic group.

Using the chi-square test, we analyzed gender differences in FE. The results indicate a statistically significant difference in awareness of the term FE, *χ*^2^ (1, *N* = 514) = 7.81, *p* < 0.05, suggesting that women are *more informed about family education programs*. There is also a statistically significant difference in the *willingness of women to attend FE programs* compared to men, *χ*^2^ (2, *N* = 513) = 12.92, *p* < 0.05. A high percentage of men answered “I don’t know”, which indicates their uncertainty. The analysis showed that women are statistically significantly *more supportive of the FE program being a legal requirement before marriage or a common-law union*, *χ*^2^ (2, *N* = 508) = 14.17, *p* < 0.05.

Furthermore, the analysis did not show a statistically significant difference in whether the state/municipality provides free FE programs, *χ*^2^ (2, *N* = 511) = 1.48, *p* > 0.05. A high percentage of men and women believe that this is an obligation of state institutions. As expected, given the lack of FE programs, a small proportion of respondents attended such programs (6% of males and 13% of females), and there was no statistically significant difference in attendance by gender, *χ*^2^ (1, *N* = 514) = 3.04, *p* > 0.05.

The results in detail regarding the gender perspective of FE are presented in Table 6.

**Table 6:** Gender perspective of FE.

Statement	Yes				No				I do not know				Chi-square
	*m*		*f*		*m*		*f*		*m*		*f*		
	*N*	%	*N*	%	*N*	%	*N*	%	*N*	%	*N*	%	
Have you heard of the term “family education”?	60	76	382	88	19	24	53	12	NA	NA	NA	NA	*χ*^2^ (1, *N* = 514) = 7.81 *p* < 0.05 significant
Would you participate in an FE program if it were available to you?	47	59	329	76	7	9	11	2	25	32	94	22	*χ*^2^ (2, *N* = 513) = 12.92 p < 0.05 significant
Should the state/municipality provide free FE programs?	77	98	417	96	1	1	2	1	1	1	13	3	*χ*^2^ (2, *N* = 511) = 1.48 *p* > 0.05 not significant
Should completing a FE program be a legal requirement before marriage or a common-law union?	34	44	285	66	25	32	76	18	18	24	70	16	*χ*^2^ (2, *N* = 508) = 14.17 *p* < 0.05 significant
Have you ever participated in an FE program?	5	6	58	13	74	94	377	87	NA	NA	NA	NA	*χ*^2^ (1, *N* = 514) = 3.04 *p* > 0.05 not significant

Based on their experience, the professionals who are part of the mapped institutions mostly (57%) confirm that there is no difference in the need for FE among different demographic groups, further 19% of them believe that they are more often visited by adolescents and people from urban areas, and 5% of the professionals highlighted middle-aged women as more frequent users of their services. Results are presented in Figure 3.

**Figure 3:**
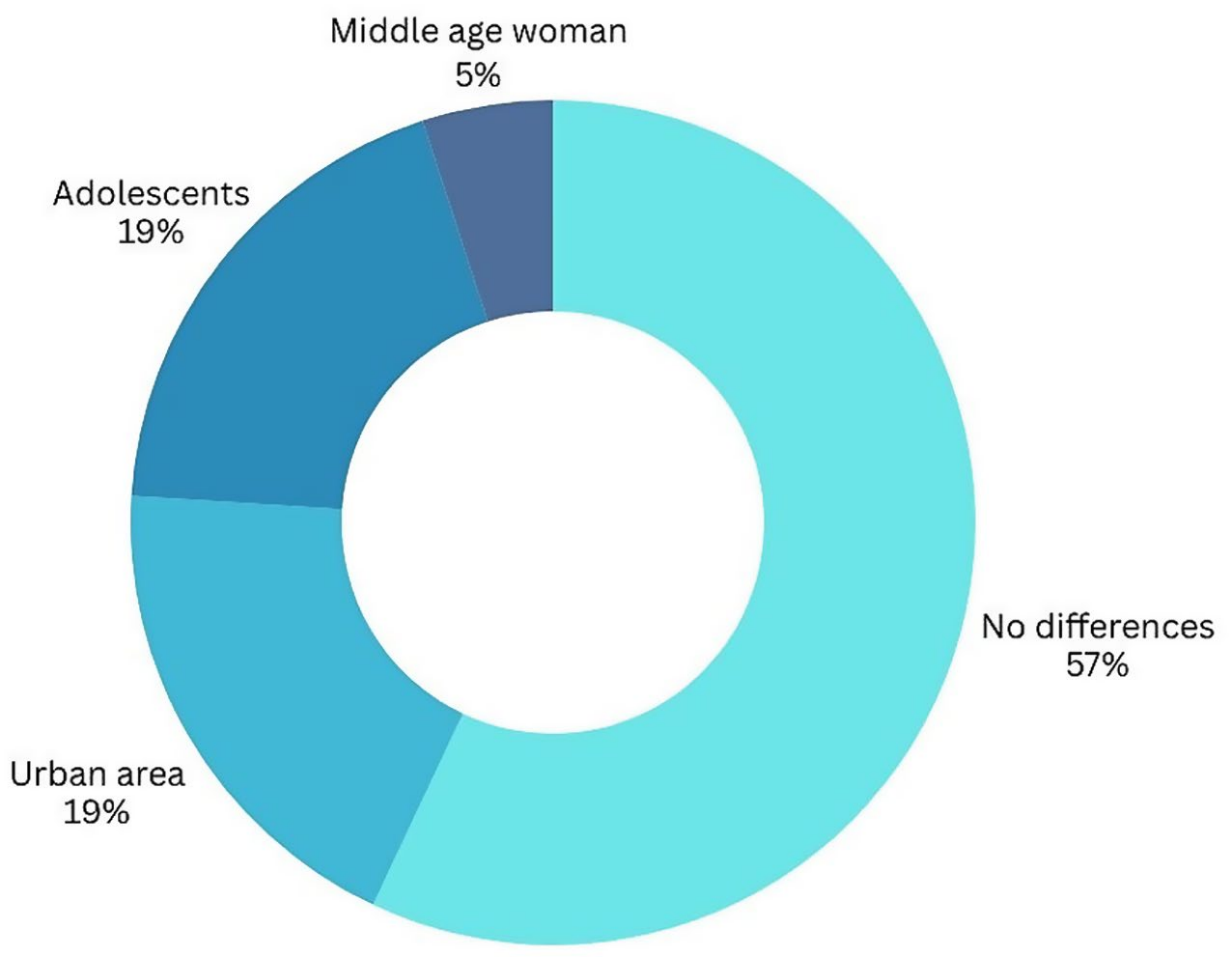
Professional perspective on demographic differences regarding the need for FE.

### 3.4 Barriers to seeking FE before divorce

The analysis of the reasons why spouses do not seek FE before serious marital problems arise (Table 7) indicates that the most common reason is the lack of available programs, with the lowest standard deviation observed (*M* = 4.11, SD = 0.84, SEM = 0.04). The impact of social stigma and shame (*M* = 3.81, SD = 1.00, SEM = 0.04) and financial challenges (*M* = 3.65, SD = 1.06, SEM = 0.05) is assessed as moderate. Respondents consider that lack of time and busyness is not a significant reason influencing the decision to attend FE programs (*M* = 3.08, SD = 1.21, SEM = 0.05).

**Table 7:** Perception of the barriers to seeking FE before divorce (survey participants’ perceptions).

Statement	*M*	SD	SEM	Cronbach’s *α*
Couples do not seek FE before divorce due to stigma or shame	3.81	1	0.04	0.92
Lack of awareness of available programs for FE	4.11	0.84	0.04	
The cost of FE	3.65	1.06	0.05	
Lack of time or busyness	3.08	1.21	0.05	

According to the analysis of the professionals’ results (Figure 4), the lack of FE programs again emerges as the primary problem (80%), followed by stigma and shame (50%). Less significant are the lack of awareness of FE’s importance (25%) and its cost (20%). Lack of time and busyness are assessed as the least relevant barriers to using FE programs (5%). Additionally, the professionals emphasized the barriers they face in providing services, noting that resource constraints (50%) are the most significant, particularly shortages of professional staff and limited space. A small proportion of respondents (5%) face a lack of training and motivation.

**Figure 4:**
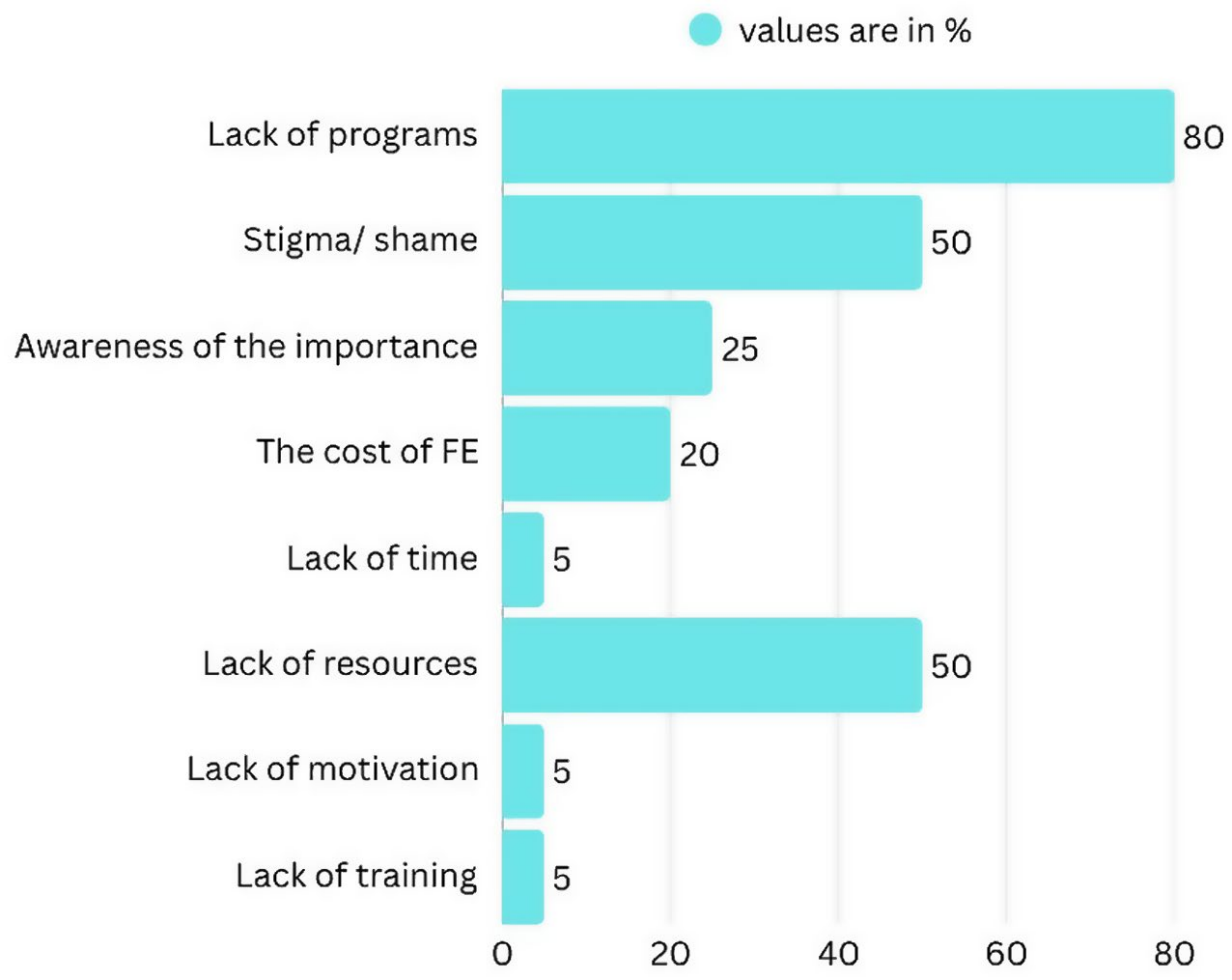
Professional perspective on barriers to seeking and providing FE programs.

## 4 Discussion

The present findings indicate a gap between the high perceived importance of FE among survey respondents and its limited institutional implementation in the RNM. Respondents perceive FE as a potentially valuable preventive and developmental intervention, especially in the premarital phase and in preserving marital communication. These results are consistent with international research confirming that partner education programs improve communication and reduce the risk of marital distress (Carlson et al., 2020; Stanley and Rhoades, 2009). Additionally, longitudinal studies show that couples who participate in premarital education have a lower probability of divorce and a higher level of marital satisfaction over a longer period (Stanley et al., 2006). These findings from previous research are consistent with respondents’ perceptions of the potential preventive role of FE, both in preventing divorce and in reducing the intensity of conflict. However, low estimates of FE availability in the Macedonian context indicate an institutional gap. This situation corresponds to the reports of the Institute for Social Affairs, which note a continuous increase in divorce counseling, regulation of personal relations with a child, and deprivation of parental rights (Бинички Арсеновски, 2024; Бинички Арсеновски et al., 2024). The increased number of interventions in already escalated conflicts suggests that the system functions predominantly reactively. In the absence of a universal preventive framework, the institutional burden shifts to crisis management, a characteristic of underdeveloped preventive systems.

The results of the research indicate that FE is perceived as a universal need, and not intended for specific groups. This perception aligns with the theory of universal prevention (Gordon, 1983) and with empirical findings confirming the long-term effectiveness of universal programs (Cinà et al., 2024; Salari and Enebrink, 2018). Within this study, FE programs appeared to be more frequently used in urban areas and by a specific group; these findings do not indicate that this group has a greater need for FE programs, but rather suggest the centralization of services and greater awareness of these services among a specific group of citizens.

The significance of the results stems from the consensus among the survey respondents and professionals that the lack of programs and institutional support for FE is the main barrier, while individual factors, such as time constraints and financial challenges, are of secondary importance. Stigma represents a significant barrier and points to the need for a cultural reframing of FE, normalizing it as part of family development rather than a secondary intervention related to the emergence of dysfunctional family patterns. Similar results were obtained by Hawkins and Erickson (2015), who indicate that a key problem in the spread of FE programs is the institutional limitations and the programs’ visibility, rather than partners’ interest.

Demographic trends point to a gradual transformation of traditional family structures, reflected in the dominance of nuclear families and the reduction in the number of family members. Within the present sample, gender-based analyses revealed statistically significant differences in several aspects of FE: women reported greater awareness of FE, greater willingness to participate in FE programs, and stronger support for their potential mandatory implementation. At the same time, both genders show similar views regarding the public provision of FE and the importance of seeking support during marital crises. Given the substantial gender imbalance in the sample, these findings should be interpreted as exploratory associations rather than as evidence of broader gender patterns in the population. However, they may be considered in relation to previous research on gender patterns of emotional labor and help-seeking. From the perspective of emotional labor theory, women have historically been described as assuming a greater share of relational and emotional work within families, including conflict regulation and relational coordination (Hochschild, 1983).

In a broader context, the present findings may reflect a pattern observed in transitional or traditionally oriented societies, where there is social consensus and legitimacy but no institutional support. Hence, in this demographic context of the RNM, marked by a decrease in population and an increase in divorces, the institutionalization of FE, as well as the development of these programs, can be understood as a social investment that will affect broader social stability in the long term. Investing in FE may strengthen families, build a more resilient community, and improve the efficiency of social protection systems.

## 5 Conclusion

The results indicate that FE is perceived by the surveyed respondents as an important form of preventive and developmental support, while in practice, it remains underdeveloped and insufficiently integrated into the institutional system. Respondents recognize its value, especially in the pre-marriage phase and during periods of marital tension, indicating a high level of receptiveness to this type of intervention within the surveyed sample. However, such support is not accompanied by an appropriate structural framework or by equal access to programs.

Data from institutions also confirm that most activities are implemented in situations of already existing conflict – divorce proceedings, regulation of relations with a child, and other complex family issues. This indicates that the system predominantly reacts when problems have already escalated, rather than taking steps to prevent them early. Such an orientation creates additional institutional pressure and limits the possibility of long-term, sustainable effects on family stability. In that sense, FE needs to be seen as a developmental resource for society. It is a mechanism for strengthening relational ties, partner responsibility, and parental competencies. FE has the potential to contribute to long-term family stability and children’s well-being.

In addition to the practical implications, the research also contributes to the development of theoretical perspectives on FE. First, it is part of the pioneering research on the state of FE in RNM by integrating respondents’ perception and institutional mapping. Second, the research examines FE in a transitional context, revealing a mismatch between the high acceptance of FE among surveyed respondents and its limited institutional implementation. Finally, the study highlights the awareness of FE from a gender perspective, pointing to the need to create family prevention systems that are more sensitive to gender roles. These findings contribute to comparative research on the institutional development of FE in emerging welfare systems.

The findings open space for a different positioning of FE within public policies. Instead of treating it as a service for specific cases or risk situations, FE should be recognized as a universal preventive measure, available at different stages of the family life cycle. In particular, mandatory FE should not be understood as a restrictive measure, but rather as educational and developmental support, aimed at strengthening relational competencies and reducing the risk of early marital conflicts. Developing a coordinated system that connects premarital education, marriage-strengthening programs, parental support, and post-divorce co-parenting education would represent a significant step towards moving from a reactive to a proactive model of support. In the context of demographic changes, rising divorce rates, and the transformation of family structures, investing in systemic prevention should not be seen as an additional cost but as a long-term social investment.

## 6 Limitation of the study

Several limitations should be considered when interpreting the findings. First, although the content validity of the questionnaires used was addressed through expert review, no formal construct validation or convergent validity testing was conducted. Second, the use of an online convenience sample may have introduced selection bias, particularly favoring respondents with higher digital literacy and interest in family-related topics. Third, the sample is predominantly composed of women with higher levels of education, which may influence perceptions of the importance of preventive education and limit generalizability to the broader population. Finally, reliance on self-report measures introduces the possibility of social desirability bias, especially in questions about marital stability and preventive responsibility.

Future research should validate the instrument and aim to include more representative sampling strategies and longitudinal designs to assess the long-term effectiveness and institutional integration of family education programs within the national welfare framework.

## Data Availability

The raw data supporting the conclusions of this article will be made available by the authors, without undue reservation.
